# Mechanisms of Radiation Toxicity in Transformed and Non-Transformed Cells

**DOI:** 10.3390/ijms140815931

**Published:** 2013-07-31

**Authors:** Ronald-Allan M. Panganiban, Andrew L. Snow, Regina M. Day

**Affiliations:** Department of Pharmacology, Uniformed Services University of the Health Sciences, Bethesda, MD 20814-4799, USA; E-Mails: ronald-allan.panganiban@usuhs.edu (R.-A.M.P.); andrew.snow@usuhs.edu (A.L.S.)

**Keywords:** ionizing radiation, apoptosis, necrosis, senescence, autophagy, cancer, primary cell

## Abstract

Radiation damage to biological systems is determined by the type of radiation, the total dosage of exposure, the dose rate, and the region of the body exposed. Three modes of cell death—necrosis, apoptosis, and autophagy—as well as accelerated senescence have been demonstrated to occur *in vitro* and *in vivo* in response to radiation in cancer cells as well as in normal cells. The basis for cellular selection for each mode depends on various factors including the specific cell type involved, the dose of radiation absorbed by the cell, and whether it is proliferating and/or transformed. Here we review the signaling mechanisms activated by radiation for the induction of toxicity in transformed and normal cells. Understanding the molecular mechanisms of radiation toxicity is critical for the development of radiation countermeasures as well as for the improvement of clinical radiation in cancer treatment.

## 1. Introduction

In Germany, 1895, Wilhelm Conrad Röntgen observed that invisible rays generated in a vacuum tube induced fluorescence from a barium platinocyanide-coated plate. He found that the rays could differentially penetrate objects of differing densities, and Röntgen generated the first radiographic images of bones. Later that same year, he reported his discovery in which he called these images X-rays, and later was awarded the Nobel Prize for Physics in 1901 [1,2]. Based on these findings, Antoine Henri Becquerel began investigations to determine whether other forms of naturally occurring phosphorescence were related to X-rays; in 1896 Becquerel discovered spontaneous radioactive properties of uranium ore, for which he too was awarded the Nobel Prize for Physics in 1903 [3]. Investigations conducted by Marie Curie on radioactive substances and their properties led to the discoveries of polonium and radium, which resulted in the receipt of Nobel Prizes in Physics in 1903 (shared with Becquerel and Pierre Curie) and in Chemistry 1911 [4]. The application of radiation has advanced the fields of medicine, biology, physics, astronomy, materials science, engineering. Indeed, the full extent of radiation application is still waiting to be realized.

Despite the many practical uses of ionizing radiation (IR), exposure to high levels of radiation has lethal consequences. The medical effects of radiation exposure have been classified into Acute Radiation Syndrome (ARS) and the Delayed Effects of Acute Radiation Exposure (DEARE). ARS consists in a constellation of health effects ensuing from an exposure of the total body or a significant area of the body to a relatively high dose (>2–5 Gy) delivered at a relatively high dose rate [5]. ARS is usually divided into three types of syndromes, based on the radiosensitivity of the organs/tissues involved: the Hematopoietic Syndrome (occurring after exposure to 0.7–10 Gy), the Gastrointestinal Syndrome (usually greater than 10 Gy), and the Cardiovascular/Central Nervous System Syndrome (>50 Gy) [5,6]. DEARE, on the other hand, consists of syndromes occurring months or years following radiation exposure which include: prolonged gastrointestinal effects, delayed immune reconstitution, delayed skin injury, renal failure, and radiation-induced lung injury [7]. Damage to normal tissue is often a limiting factor for the clinical use of radiation for cancer eradication; all forms of cell death as well as accelerated senescence have been linked to delayed cycles of inflammation, tissue dysfunction, atrophy, and/or fibrotic remodeling [8–12]. While there has been an exponentially growing number of studies attempting to explain the mechanisms of radiation toxicity over the years, there are only a limited number agents approved by the Food and Drug Administration as countermeasures for prevention, mitigation, or treatment of ARS or DEARE [13].

## 2. Ionizing Radiation-Induced Damage to Biological Molecules

Ionizing radiation (IR) generates both direct and indirect damage to biological molecules. In high linear energy transfer (LET) radiation, such as neutrons and alpha particles, most of the cellular damage results from the direct ionization of cellular macromolecules including DNA, RNA, lipids, and proteins [14]. In contrast, low LET radiation, such as X-rays and gamma rays, indirect damage to biological macromolecules occurs following the generation of reactive oxygen species (ROS) [14]. ROS, especially superoxide and hydroxide radicals from the radiolysis of intracellular H_2_O, can have many effects, including the oxidation of biological macromolecules and activation of intracellular signaling pathways [14–18].

A widely accepted dogma in the field of radiation biology is that DNA is the most important molecular target of radiation because of its critical role in cell replication and proliferation [19]. Both single-stranded DNA breaks (SSB) and double-stranded DNA breaks (DSB), along with nucleotide mutations, occur during IR exposure and lead to cell death or mutagenesis if not properly repaired [20,21]. DSB have more lethal consequences than SSB, as noted in low LET radiation when DSB are induced in a relatively large fraction of cells [20]. Overall, radiation-induced DNA damage is believed to activate a variety of signaling pathways leading to cell death as well as accelerated senescence.

Recent investigations have challenged the classical DNA-centric view of radiation injury by demonstrating that proteins are also critical radiation targets that influence cell death mechanisms. In some cases, radiation-induced death by protein damage is proposed to be a consequence of reduced DNA repair fidelity, indirectly decreasing cell viability [22]. In prokaryotes, Qui *et al.* [23] suggested that protein damage underlies the radiosensitivity of *Shewanella oneidensis* while Daly *et al.* [24] proposed that the extreme radioresistance of *Deinococcus radiodurans* has been attributed to the reduction of protein oxidation by a variety of protective mechanisms. *Bdelloid* rotifers also display resistance to radiation damage due to decreased protein oxidation [25]. Studies using cultured mammalian cells have also provided evidence for protein oxidation in the activation of pro-apoptotic signaling downstream of radiation damage [26,27]. However, a direct comparison has not yet been made for the contribution of protein damage *versus* DNA damage for overall cellular toxicity.

## 3. Ionizing Radiation-Induced Cell Toxicities

The molecular mechanisms of radiation-induced cellular injury depend on a number of factors including radiation dosage, the cell type, and the transformed status of the cell [21,28,29]. As suggested by the manifestation of acute and delayed radiation syndromes, specific tissues and organ systems have differential radio-sensitivity. In several cases, the vulnerability of tissues to radiation injury is predicted by the Law of Bergonie and Trebondeau which states that radiation is generally more damaging in rapidly dividing cells and in undifferentiated cells [28,30]. For example, untransformed epithelial cells of the gastrointestinal tract and progenitor cells of the hematopoietic system, which have rapid turnover rates, are generally more radiosensitive than the non-dividing neurons of the central nervous system. This differential proliferative capacity corresponds to the induction of Hematopoietic Syndrome at lower radiation exposures (0.7–10 Gy) compared to doses required for inducing Central Nervous System Syndrome (>50 Gy).

Unrepaired DNA damage can lead to mutations, genomic instability, and cell death. Cells have evolved complex systems for the repair of single- and double-stranded DNA breaks [31]. It has been demonstrated that normal (non-transformed, non-immortalized cells) can repair as many as 70 DSB/cell within 24 h of radiation exposure [32]. Different DNA repair mechanisms are thought to be activated during specific phases of the cell cycle [28,33]. DSB can be repaired via a homologous recombination-dependent mechanism during the G2/M phases of the cell cycle, whereas non-homologous end joining mechanisms are believed to be active during G1/G0. In contrast, DNA repair is relatively inefficient during the S phase of the cell cycle [28]. Importantly, the duration for activity of a particular DNA repair mechanism depends upon the time that the cell remains in a particular phase of the cycle [28]. Therefore, cells that move rapidly through the cell cycle have less time to repair their DNA than cells that are paused during a cycle in which a particular DNA repair mechanism is activated.

Our current understanding of the mechanisms of ionizing radiation-induced cell death comes from studies that are mostly conducted on immortalized cancer cell lines that do not represent the biological status of non-immortalized, non-transformed normal cells [29]. Although cancer cells proliferate more quickly than normal cells, leaving their DNA more susceptible to unrepaired damage, these cells often contain multiple mutations resulting in constitutive activation of mechanisms for DNA repair or allowing them to survive following damage that would render normal cells unviable [34].

**Figure 1:**
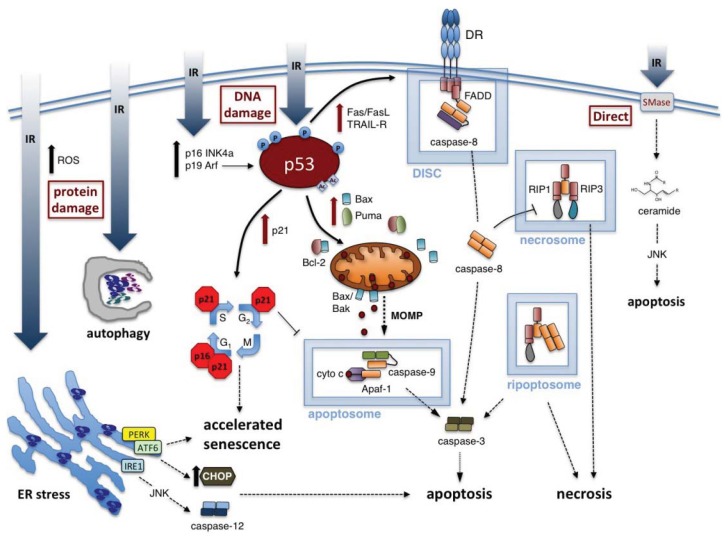
Molecular responses to ionizing radiation (IR) in exposed cells. Depending on dose and radiosensitivity of the exposed cell, IR may induce cell death (through apoptosis or necrosis) or trigger accelerated senescence. Increased expression of p53, coupled to various post-translational modifications (e.g., phosphorylation (P), acetylation (Ac)), is a critical step in mediating the cellular response to IR-induced DNA damage. Accelerated senescence can result from p53-dependent induction of p21/waf1 or upregulation of other cell cycle inhibitory proteins (e.g., p16 INK4a). p53 activation also triggers *de novo* synthesis of pro-apoptotic molecules that mediate intrinsic (e.g., Bax, Puma) or extrinsic (e.g., Fas) apoptotic cell death (red arrows, p53-dependent). Intrinsic apoptosis is governed by Bcl-2 family proteins that regulate mitochondrial outer membrane permeabilization (MOMP), whereas extrinsic apoptosis is signaled through dedicated death receptors (DRs) such as Fas. Both forms of apoptosis rely on the assembly of large multiprotein platforms, including the apoptosome and death-inducing signaling complex (DISC), which facilitate caspase activation through recruitment, dimerization and autocatalytic cleavage. Separate protein complexes containing RIP-1 and/or RIP-3 (e.g., necrosome) can trigger programmed necrosis under certain conditions (e.g., caspase-8 inhibition). IR can also elicit ER stress and autophagy in response to the accumulation of oxidized or misfolded proteins, which may in turn induce apoptosis. Finally, apoptosis may also be triggered by increased ceramide levels, generated through direct IR-induced activation of sphingomyelinases (SMases) in the plasma membrane.

Radiation exposure to cells has been demonstrated to result in a variety of mechanisms of cell death, including necrosis, apoptosis, or autophagy (see Figure 1) [35]. Additionally, radiation may induce accelerated cellular senescence, a state in which the cell remains viable but with altered functions, and which is no longer competent for proliferation [36]. In some cases, it has been demonstrated that increasing IR dosages shift the cellular response from senescence to apoptosis and/or autophagy, with higher doses leading to necrosis [27]. However, there is no absolute response of all cells to a given dose of radiation exposure. Some cell types rapidly undergo apoptosis in response to the same level of radiation that induces senescence in another cell type (e.g., primary human hematopoietic CD34^+^ cells undergo apoptosis whereas pulmonary artery endothelial cells primarily undergo accelerated senescence) [27,37]. The selection process resulting in a specific mode of cell death or senescence has not been clearly defined, but research indicates that it is affected by the radiation dose, the dose rate, and multiple aspects of the cellular context [31,32,34,38,39].

### 3.1. Radiation-Induced Apoptosis

Apoptosis, or programmed cell death, is an evolutionarily conserved and highly regulated form of cell death required for the removal of extraneous, damaged, infected, or transformed cells from normal tissues [40]. Apoptosis is characterized by chromatin condensation, DNA fragmentation, cell shrinkage, and ultimately disintegration of the cell into membrane-bound particles [41,42]. These apoptotic “blebs” are rapidly removed by phagocytic cells *in vivo* to prevent cell death-associated inflammation. Two principal apoptotic pathways are widely recognized—extrinsic apoptosis and intrinsic apoptosis. The extrinsic pathway is activated by extracellular signals transduced by transmembrane “death receptors”. In contrast, the intrinsic pathway is initiated by signaling pathways from inside the cell that govern mitochondrial integrity. The use of either of these pathways depends on the nature and origin of the death signal [43,44]. Each apoptotic pathway regulates the activation of specific initiator caspases, a family of cysteine-aspartic proteases required for apoptotic cell death signaling [45]. The two apoptotic pathways eventually converge with the activation of central activator caspases (e.g., caspase-3, -6, and -7) required for proteolytic processing of key cellular proteins as well as DNA fragmentation [45].

Many cancer cells, including lung, prostate, immortalized keratinocytes, and colon cancer cells, commit to apoptotic cell death when exposed to radiation ranging from 1 to 20 Gy [31,46–49]. Low doses of radiation (10–200 cGy) have been demonstrated to induce apoptosis in human skin organotypic culture and murine epidermal cells [50]. In response to low to moderate doses of radiation (≤32 Gy), neurons also undergo apoptosis *in vitro* [11]. In contrast, some non-transformed, non-immortalized cells, such as smooth muscle cells and pulmonary artery endothelial cells, display apoptotic responses only when exposed to higher doses of radiation (>20 Gy) [27,51]. One important characteristic of apoptosis that it is generally non-inflammatory, with reduced effects on neighboring cells compared to other modes of cell death [52]. However, tumor cell apoptosis has been linked to the induction of increased tumor cell growth in some studies [53]. The mechanisms of apoptotic cell death induced by IR are further described below.

#### 3.1.1. Intrinsic Apoptosis Induced by IR

Because IR is known to cause DNA damage that can initiate a variety of intracellular signaling, the intrinsic pathway has been inferred to be the primary apoptotic mechanism mediating IR-induced apoptosis. Intrinsic apoptosis, also referred to as the mitochondrial pathway, is characterized by mitochondrial outer membrane permeabilization (MOMP) and cytochrome c release [54,55]. Cytosolic cytochrome c interacts with apoptotic protease activating factor 1 (Apaf1) and procaspase-9, forming the apoptosome [43,56]. The primary function of the apoptosome is to activate the initiator caspase-9, which triggers a cascade of caspase activation beginning with the executioner caspase 3 [56,57].

Intrinsic apoptosis is initiated by signaling pathways activated in response to DNA damage [6,31]. DNA breaks are initially sensed by several groups of proteins including: the mediator of DNA damage checkpoint protein 1 (Chk1); p53 binding protein 1 (53BP1); DNA-dependent protein kinase (DNA-PK); the protein complex meiotic recombination 11 homolog 1, Rad50 protein, and nibrin (Mre11/Rad50/NBS1, MRN); and the protein complex Rad9, Rad1, and Hus1 (Rad9/Rad1/Hus1, 9-1-1) [31,33]. Binding of MRN and 9-1-1 to DNA leads to the activation of a number of kinases that amplify the DNA damage response, including the Ataxia telangiectasia mutated protein (ATM) kinase and the ATM and Rad3-related (ATR) kinase [31,35,58]. DNA-PK, ATM and ATR phosphorylate the tumor-suppressor protein p53, the critical mediator of DNA damage-induced intrinsic apoptosis [35,39,57,59–61].

p53 is referred to as the “guardian of the genome” because of its role in maintaining chromosomal stability and determining the fate of the cell following DNA damage: either survival with temporary cell cycle arrest (during which damaged DNA is repaired) or apoptotic cell death [33,44,62]. Expression of p53 protein increases immediately following DNA damage. This increase in p53 protein levels has been shown to be proportional to the extent of DNA damage in a cell, and the kinetics of p53 regulation vary with different types of radiation [63]. Specific posttranslational modifications of p53 (phosphorylation and acetylation) stabilize the protein and activate its functions as a transcription factor as well as modulate its association with other proteins [64]. For example, CREB (cAMP response element-binding) binding protein (CBP)/p300 and the p300-CBP-associated factor (PCAF) acetylate p53, protecting it from ubiquitination and degradation [64]. Acetylation and phosphorylation are also required for p53 transcriptional activity and changes in protein-protein interactions [65,66]. Depending upon the extent and nature of p53 activation, divergent pathways may be activated [63].

p53 dictates apoptosis sensitivity in part through the regulation of levels and functions of pro-apoptotic and anti-apoptotic proteins of the B-cell lymphoma 2 (Bcl-2) protein family [67]. The anti-apoptotic members of the Bcl-2 family protect the mitochondria from MOMP and cytochrome c release, primarily by binding to and neutralizing Bax and Bak (two pro-apoptotic members of the Bcl-2 family), which directly facilitate mitochondrial permeabilization. There are three primary effects of p53 on this system: 1) Increased expression of pro-apoptotic Bcl-2 family proteins; 2) disruption of the binding between pro- and anti-apoptotic Bcl-2 family proteins; and 3) induction of pro-apoptotic Bcl-2 protein oligomerization. p53 nuclear localization and transcriptional activation leads to increased gene expression of pro-apoptotic Bcl-2 family proteins such as Bax and Puma [68–72]. Furthermore, p53 in the cytoplasm can localize to the mitochondria, where it inhibits the association between anti-apoptotic proteins Bcl-2 and Bcl-x_l_ with the pro-apoptotic Bax and Bak proteins [35,59,73]. Note that in contrast with its function in the activation of mitochondrial apoptosis, p53 is not localized to the mitochondria during the induction of cell cycle arrest [67]. A combination of release of Bax from Bcl-2-protein binding as well as direct interactions with p53 lead to the oligomerization of Bax and Bak, which then form channels in the mitochondrial membrane inducing MOMP, cytochrome c release, and subsequent apoptosome formation [43,56,74].

The expression level and mutational status of p53 can affect the cellular decision to undergo apoptosis. Studies have indicated that tissues sensitive to radiation-induced apoptosis (spleen, thymus, and testis) display more rapid increases in p53 proteins compared with radioresistant tissues (liver and kidney) [67]. Additionally, cancer cells may evade radiation-induced apoptosis by suppression of p53 expression, or by gaining mutations in p53 that nullify its pro-apoptotic activity [75]. Indeed, over 50% of all human tumors harbor mutations or deletions in the *TP53* gene [76]. Thymocytes lacking p53 were shown to be more resistant to apoptosis induced by DNA DSB than those having wild-type p53 [77,78]. Overexpression of wild-type p53 increases apoptosis in p53-deficient leukemic and colon tumor-derived cell lines [79,80]. A number of agents that increase p53 levels are currently being utilized to increase apoptosis sensitivity of tumor cells to radiation therapy [67].

Independently of p53 function, the sphingomyelin pathway also mediates radiation-induced apoptosis through ceramide generation. Ceramide is an important second messenger molecule for regulating stress responses, including apoptosis induction [81]. For example, IR exposure in endothelial cells has been shown to activate sphingomyelinases (SMases) that hydrolyze sphingomyelin in the plasma membrane to generate ceramide. In fact, mouse embryonic fibroblasts (MEFs) from acid SMase knockout mice are completely resistant to radiation-induced apoptosis [82]. Ceramide appears to engage multiple modes of apoptotic signaling (e.g., JNK activation, PI-3K inactivation, *etc.*) that ultimately require MOMP, even when the death receptor CD95/FAS is implicated [81]. Ceramide can also stimulate BAX integration into the mitochondrial outer membrane to directly facilitate MOMP [83]. Although more studies are needed, pharmacological manipulation of ceramide may represent an attractive and relatively accessible strategy for modulating cellular responses to IR.

#### 3.1.2. Extrinsic Apoptosis Induced by IR

In contrast with the intrinsic apoptotic pathway, the extrinsic pathway of apoptosis is initiated by extracellular activation of transmembrane “death” receptors (DRs)—CD95/Fas, tumor necrosis factor receptor 1 (TNF-R1), and tumor necrosis factor-related apoptosis-inducing ligand (TRAIL) receptors DR4 and DR5—by their corresponding ligands [43,44]. These DRs contain conserved intracellular “death domains” which, upon ligand binding, cluster and nucleate the death-inducing signaling complex DISC containing the adapter protein Fas-associated death domain (FADD) protein and procaspase-8 and -10 [44,62]. Caspase-8 is the main initiator caspase of the extrinsic apoptotic pathway, equivalent to caspase-9 in the intrinsic pathway. Similar to caspase-9 in the apoptosome, procaspase-8 undergoes autocatalytic cleavage within the DISC, and the active form of caspase-8 directly cleaves and activates the executioner caspases-3 and -7 [84].

*In vivo* studies have demonstrated that mice deficient in extrinsic apoptosis signaling have reduced apoptosis in response to radiation in a tissue-specific manner. In response to IR, *DR5* knockout mice show reduced apoptosis in the thymus, spleen, Peyer’s patches, and the white matter of the brain, with normal levels of apoptosis in the ileum, colon, and stomach [85,86]. IR also causes caspase-8-mediated apoptosis in Jurkat T cells, glioma cells, and some breast cancer and human lymphoma cell lines [87–90]. However, in a number of instances, the activation of both intrinsic and extrinsic apoptosis has been observed simultaneously. Some cancer cells exhibit both extrinsic and intrinsic apoptosis in response to IR [91]. Our laboratory also recently demonstrated that in normal lung endothelial cells caspase-8 is activated by IR concurrent with the activation of caspase-9 [27]. The distinction between intrinsic and extrinsic apoptosis can become blurred, since the effector caspases 3 and 7, which are activated during both intrinsic and extrinsic apoptosis, can feedback to regulate mitochondrial events, such as Bax translocation and cytochrome c release [92].

Several mechanisms have been proposed for the regulation of the extrinsic apoptotic pathway by IR. In some tissues, the regulation of DRs following DNA damage may proceed through the activation of p53 transcriptional activity [31,85]. p53 has been demonstrated to upregulate TRAIL receptors and CD95/Fas, as well as the Fas ligand [85,93]. However, in some tissues, such as the thymus and colon, the regulation of death receptors was shown to be p53-independent [94,95].

Interestingly, the specific inhibition of extrinsic apoptosis is a common mechanism for cancer cell evasion of cell death signals. For example, various isoforms of cellular FLICE inhibitory protein (cFLIP) contribute to DR-induced apoptosis resistance by interfering with caspase-8 activation at the DISC [96]. Therapeutic strategies for targeting cFLIP to lower the threshold for DR-induced apoptosis are generating considerable interest for cancer therapy [97]. Inhibitor of apoptosis proteins (IAPs) also modulate apoptosis and necrosis sensitivity through their ubiquitin ligase activity (e.g., c-IAP1/2) or by directly inhibiting certain caspases (e.g., XIAP) [98]. Apoptosis repressor with caspase recruitment domain (ARC) also functions to suppress both intrinsic and extrinsic apoptosis through multiple mechanisms, including the inhibition of caspase activation, the inhibition of association of DRs with downstream signaling molecules, and through direct inhibition of Bax [99]. Like IAPs, ARC has been found to be overexpressed in a variety of cancer cells, including glioblastoma, melanoma, and lymphoma cells, and in cancers from pancreas, colon, breast, lung, cervix and prostate [100]. Paradoxically, the overexpression of anti-apoptotic Bcl-2 proteins can also protect cancer cells from extrinsic apoptosis, when MOMP and caspase-9 activation are required to amplify the DR-induced caspase cascade [101–103]. Additional mechanisms have been identified for cancer cell evasion of the extrinsic pathway, including downregulation of DRs and the suppression or mutation of proteins required for death receptor signaling [104–107]. Not surprisingly, proteins regulating extrinsic apoptosis have become therapeutic targets for improving the efficacy of both clinical radiation and chemotherapy in resistant cancer cells [102,107–109].

#### 3.1.3. ER Stress and Activation of Apoptosis by IR

DNA damage is a critical target for IR-induced cell toxicity, but increasing evidence indicates that protein damage also contributes significantly to cell death in some organisms [23–25]. In eukaryotic cells, the endoplasmic reticulum (ER) serves as a sensor of cellular homeostasis, being the site for protein folding and maturation for secretory and membrane proteins [110]. Specifically, the ER initiates signaling in response to damaged proteins. An accumulating body of evidence implicates oxidized, damaged proteins as triggers of the ER stress pathway (also known as unfolded protein response, UPR) [111,112]. A critical increase in the level of unfolded proteins is detected by the ER. The ER stress response pathway is designed to transiently halt new protein synthesis and increase the expression of chaperones to promote protein folding as well as to induce the ER-associated degradation (ERAD) system to remove terminally damaged proteins from the ER for proteasome-dependent degradation [110]. Failure to eliminate the damaged/unfolded proteins from the ER can ultimately result in apoptosis or accelerated senescence [110,113,114]. Protein unfolding may typically activate three ER-localized sensors: (1) the RNA-like endoplasmic reticulum kinase (PERK); (2) the activating transcription factor 6 (ATF6); and (3) the inositol-requiring enzyme 1 (IRE1) [112]. PERK, ATF6 and IRE1 are bound in the ER lumen and held in an inactive state by the chaperone glucose-regulated protein 78 (GRP78). Release of these proteins from GRP78 is believed to be the initiating event in UPR [115].

PERK, a transmembrane protein resident of the ER, phosphorylates the eukaryotic initiation factor 2 alpha (eIF2α), leading to the inhibition of protein synthesis to reduce the traffic of newly-synthesized, unfolded polypeptides to the ER [116,117]. Although eIF2α phosphorylation inhibits most protein synthesis, phosphorylated eIF2α specifically enhances some translation, including the translation of the ATF4 transcription factor that can induce the expression of other genes involved in the UPR, such as chaperones, amino acid transporters, and antioxidant proteins [110].

Activation of the ATF6 transcription factor by UPR allows nuclear translocation of the factor to increase the expression of several target genes, including chaperones GRP78 and GRP94, and the transcription factor X-box-binding protein 1 (XBP1). Interestingly, XBP1 mRNA requires further processing by IRE1, suggesting that signaling cross-talk tightly regulates the ER stress pathways (see below) [110]. In some cell types ATF6 also regulates acute inflammatory response genes during ER stress, which can lead to systemic effects [118].

IRE1 is a Ser/Thr protein kinase and endoribonuclease. IRE1 cleaves a number of mRNAs including mRNAs targeted for the ER as well as the 28S ribosomal subunit; both of these events are likely involved in the reduction of protein synthesis [110]. IRE1 also initiates unconventional splicing of XBP1 mRNA in the cytoplasm, allowing XBP1 to be translated in its active form. The active form of XBP1 protein is then translocated to the nucleus where it binds to the ER stress response element (ERSE) found in the promoters of target genes, resulting in the subsequent increased expression of other chaperones, transcription factors, ER associated protein degradation components, and proteins involved in the secretory pathway [119].

In a later stage of ER stress, if protein unfolding is resolved, both eIF2α and ATF6 up-regulate the C/EBP homologous protein transcription factor (CHOP) [112,120]. CHOP targets expression of the growth arrest and DNA damage-inducible protein 34 (GADD34), a phosphatase that dephosphorylates eIF2α to restore protein translation [112]. However, if the levels of unfolded proteins have not been reduced, the activation of eIF2α by CHOP can induce further ER stress [110]. Data indicate that CHOP, c-Jun NH_2_-terminal kinase (JNK), and caspase activation are all involved in ER stress-induced apoptosis [110]. During ER stress, IRE1 can recruit TRAF2 to the plasma membrane, which leads to the activation of the JNK pathway and subsequent cell death via caspase-12 activation [121]. Other studies demonstrated that JNK activation downstream of ER stress leads to Bax activation, MOMP, and activation of caspase 3 [122].

Studies indicate that ROS and ROS-generating substances induce ER stress leading to cell death [113,123–126]. Radiation has also been shown to elicit the induction of ER stress in immortalized cell lines and in normal endothelial cells [27,127]. Our laboratory recently demonstrated that in primary lung endothelial cells IR-induced ER stress contributes to apoptosis but not to accelerated senescence [27]. Inhibition of ER stress by salubrinal reduced levels of caspase-3 activation but not levels of p21/waf upregulation in response to IR. Blockade of ER stress and JNK activation during IR exposure also inhibits MOMP and caspase activation via the intrinsic apoptosis pathway in some transformed cell types [122]. Methodologies for increasing IR-induced ER stress are currently a topic for cancer therapeutic research [128–130].

### 3.2. Radiation-Induced Necrosis

In contrast to apoptosis, necrosis is traditionally viewed as a passive process, characterized by the early rupture of the plasma membrane, dilatation of cytoplasmic organelles, and uncontrolled release of cytoplasmic contents [131,132]. Necrosis typically results from a higher magnitude of stress relative to either apoptosis or cellular senescence [31,131,133], and radiation-induced necrosis has been demonstrated *in vitro* and *in vivo* [134–137]. For example, high radiation exposures (≥ 32–50 Gy) were demonstrated to induce necrosis in neurons and in p53-deficient human leukemia cells in cell culture [11,138]. However, lower doses of gamma radiation (0.5 Gy) were shown to cause necrosis in the immortalized human keratinocyte cell line HaCaT [137]. In contrast to apoptosis*,* necrosis (including radiation-induced necrosis) is associated with increased inflammation of the surrounding normal tissue [8,139].

In contrast to necrosis induced by severe physiochemical stress, recent research has focused on programmed necrosis or necroptosis [140]. This dedicated signaling pathway is often triggered by traditional apoptotic stimuli (e.g., TNF, FasL, TRAIL) when caspase-8 activity is inhibited by deletion or binding to cFLIP and/or viral inhibitors such as CrmA or vICA, permitting the formation of “necrosome” protein complexes containing receptor interacting proteins 1 and 3 (RIP1/RIP3). Deubiquitination of RIP1 allows it to interact with the death domain of FADD, promoting recruitment and mutual phosphorylation of RIP1 and RIP3 and downstream necroptotic signaling preferentially through the mitochondrial permeability transition complex [141,142]. In response to genotoxic stress, a similar “ripoptosome” containing FADD, caspase-8 and RIP1 can assemble and mediate either apoptosis or necroptosis, independent of DRs or mitochondrial signaling [143]. In both scenarios, necroptosis is specifically blocked by the RIP1 kinase inhibitor necrostatin-1 [144]. To date, a single study has demonstrated that necroptosis involving RIP1 contributes to IR-induced cell death in irradiated anaplastic thyroid and adrenocortical cancers [145]. IR-induced cell death was blocked in those tumors by necrostatin-1 treatment, suggesting strategies to promote RIP1 activation may help to radiosensitize certain cells. However, such a strategy must weigh the risks and benefits of promoting the associated release of highly immunogenic molecules known as “danger-associated molecular patterns” (DAMPs) from necrotic cells [140]. Enhanced inflammation triggered by necroptosis may augment anti-tumor immune responses, but ultimately provoke unintended damage to healthy tissues.

### 3.3. Radiation-Induced Autophagy

Autophagic cell death is a nonapoptotic (caspase-independent), highly evolutionarily conserved mechanism of programmed cell death, characterized by the catabolism of cellular constituents by the cell’s own intracellular enzymes [146]. Under normal conditions, microautophagy and chaperone-mediated autophagy allow the limited breakdown of abnormal proteins, cellular debris, or damaged organelles to maintain cellular homeostasis and/or as a means to recycle biological components [146]. Under extreme conditions—including nutrient starvation, oxidative or genotoxic stress, protein aggregation, or extreme organelle damage—autophagy, also called macroautophagy, can result in cell death [147]. Autophagy is recognized as an independent mechanism of programmed cell death, but there is significant cross-talk between pathways activated for autophagy and apoptosis [147].

In mechanistic studies of micro- and macro-autophagy, the nucleation of the double-membraned autophagosome vesicle was demonstrated to require the formation of two key complexes, one comprised of multiple autophagy-related gene (Atg) proteins and the other containing microtubule-associated protein 1 light-chain subunit 3 (LC3) [146,147]. Nucleation of the phagophore can be initiated by the activation of phosphoinositide-3-kinase (PI3K), by mitochondrial activation of the extracellular regulatory kinase (ERK), or by the activation of c-Jun *N*-terminal kinase (JNK) [146]. The phagophore elongates through the incorporation of proteins and lipids to encapsulate the designated cargo. This process is mediated by two ubiquitin-like systems involving a number of Atg proteins and LC3 [147]; although not completely defined, phagophore elongation is hypothesized to involve soluble *N*-ethylmaleimide-sensitive factor attachment protein receptor (SNARE) proteins and endosomal trafficking proteins [146]. Once the autophagosome has matured and is sealed, it fuses with the lysosome to form the autophagolysosome, in which the cargo is degraded. The process of autophagy involves complex regulation by protein sensors of nutrient levels and metabolism, by ER stress pathways, by mitochondrial stress pathways, and by oxidative and nitrative signaling [146].

Because autophagy pathways can function to remove damaged cellular components and serve as a mechanism for programmed cell death, autophagic pathways can paradoxically allow either increased survival or cell death in response to IR depending upon the cellular context [147–149]. IR has been demonstrated to induce microautophagy or macroautophagy in a variety of cancer cells, including breast, colon, lung, esophageal, and prostate cancer cells [129,130,150,151]. Interestingly, key proteins regulating autophagy were shown to be significantly decreased in normal lung tissue *in vivo* 24 h following 6 Gy exposure, suggesting a specific and severe dysregulation of autophagy in this tissue following IR. This effect was not observed in kidney or liver tissue following identical radiation exposures [152].

Several studies have shown that in some cases, agents that increase microautophagy result in improved cellular survival from IR, possibly due to the increased clearance of damaged macromolecules that would otherwise trigger cell death. The PI3K/Akt pathway is a pro-survival pathway in normal and cancer cells, and it was hypothesized that the inhibition of PI3K/Akt in conjunction with IR would increase apoptosis. Paradoxically, the use of inhibitors of the PI3K/Akt pathway (such as Ly294002 and PI-103) actually increased survival in cancer cells exposed to IR through the microautophagy [153]. Chloroquine and bafilomycin A1, both weak bases, raise the pH of the lysosome, and block the fusion of the phagosome with the lysosome, inhibiting late stages of autophagy. In contrast, 3-methyladenine (3-MA) inhibits early autophagy by interfering with the formation of a phagophore initiation complex [146]. Inhibition of autophagy with chloroquine, balfilomycin A1, or 3-MA increased cancer cell death in response to IR, including that of cancer stem cells, via increased apoptosis triggered by the accumulation of autophagosomes [153].

In contrast with these studies, induced autophagy in some cancers results in sensitization to IR. Activation of autophagy abrogated radiation resistance of glioblastoma cells and lung cancer cells to IR [154,155]. Additionally, the zinc ionophore PCI-5002 was demonstrated to radiosensitize lung cancer cells by inducing autophagic cell death [150]. Agents for the sensitization of cancer cells to radiation-induced autophagy are currently being investigated for improving tumor responses to clinical radiation [156,157].

### 3.4. Radiation-Induced Accelerated Senescence

The concept of cellular senescence was first introduced as early as the 1960s by Hayflick and Moorhead who demonstrated that nontransformed, nonimmortalized cells were capable of a finite number of passages before they lost their ability to replicate *in vitro*; this phenomenon is known as the “Hayflick limit” [158,159]. In contrast with growth-arrested quiescent cells that may resume proliferation in response to physiological stimuli, the growth arrest of senescent cells is essentially irreversible [160]. Various stress stimuli have been shown to induce cellular senescence, including oxidative stress, DNA damaging agents such as IR and chemotherapy, and sustained signaling by certain cytokines such as interferon-alpha and transforming growth factor-β (TGF-β) [161,162]. Senescence can also be induced by oncogenic gene activation, and thus the senescence pathway may provide a mechanism by which cells avoid neoplastic transformation [162,163]. Much of our understanding regarding the activity of senescent cells is derived from cell culture studies, but the importance of senescence *in vivo* is increasingly being recognized. The accumulation of senescent cells in older tissues and in tissues following IR or chemotherapy may account for some age-associated and IR- or chemotherapy-induced pathologies [36,162,164].

Senescent cells display a wide variety of alterations in gene expression, including: (1) aberrant expression of cell cycle regulatory proteins, contributing to the inability to progress through the cell cycle; (2) upregulation of anti-apoptotic proteins; and (3) robust expression of mRNAs for secreted proteins includes inflammatory cytokines, growth factors, and proteases, resulting in a condition termed the “secretory phenotype” [36,164]. In culture, senescent cells have a notably altered morphology, and may assume a “fried egg”-like appearance, with altered cytoskeletal organization and changes in cell-cell contacts. Senescence in many cells has been demonstrated to correlate with increased expression of a form of β-galactosidase, termed senescence-associated β-galactosidase (SA-β-gal), which has become a recognized marker for senescence [165]. Two primary DNA damage-induced signaling pathways have been demonstrated to lead to cell cycle arrest associated with senescence: the p53/p21waf1 pathway, and the p16 inhibitor of cyclin dependent protein kinase 4(p16INK4a)/retinoblastoma protein (Rb) pathway [166].

Paradoxically, both accelerated senescence and apoptosis can involve stabilization and activation of the p53 protein [163]. Studies indicate that the mechanism of divergent signaling by p53 for differential activation of cell cycle arrest or cell death is not completely understood [167]. Stabilization of p53 in senescence is believed to occur in part by its acetylation as the result of ATM activation and the inactivation of NAD-dependent deacetylase sirtuin-1 (SIRT1), a deacetylase of p53 [168,169]. In contrast with apoptosis, which requires p53 regulation of apoptotic gene proteins, a critical step in p53-induced senescence is the transcriptional activation of p21/waf1. p21/waf1, a cyclin dependent kinase inhibitor 1, inhibits cyclin-dependent kinases (Cdks) including Cdk2 and 4/6; the specific phase of the cell cycle inhibited by p21 may be cell type-dependent, and blockade in G_1_, G_2_, or S-phase have been reported following DNA damage [161,167,170–173]. p21/waf also protects the cell from undergoing apoptosis following DNA damage [167,174]. The initiation of apoptosis often requires an active cell cycle, which is effectively blocked by expression of p21 [175,176]; additionally p21 can interfere with apoptotic signaling by through direct interactions with pro-apoptotic proteins, thus providing a second mechanism for the generation of cell cycle arrest instead of cell death [174,177,178]. Recently the E2F-associated phosphoprotein (EAPP) was demonstrated to be upregulated in response to DNA damage and to independently increase p21/waf1 transcription; EAPP was also shown to inhibit DNA damage-induced apoptosis in a p21/waf1-dependent manner [174].

A second pathway for DNA damage-induced cellular senescence involves regulation of p16INK4a and Rb [166,169]. IR was demonstrated to increase cellular levels of p16INK4a and the alternative reading frame protein of INK4a (p19Arf), an inhibitor of the p53 inhibitor mouse double minute 2 homolog (Mdm2) [179,180]. These two proteins act together to stop cell cycle progression. p16 INK4a is a cyclin dependent kinase inhibitor; by inhibiting Cdk kinases p16INK4a prevents the phosphorylation and inactivation of the tumor suppressor protein Rb [181]. The primary cell cycle-inhibitory activity of Rb appears to involve its interaction with the members of E2F family of transcription factors. E2F proteins regulate a number of genes involved in the progression through the cell cycle, and Rb binds to E2F proteins, sequestering them in the cytoplasm and inhibiting their transcriptional activity [181]. As its name indicates, p19Arf is transcribed from an alternative reading frame at the same locus as p16INK4a [181,182]. p19Arf is known to inhibit the cell cycle through several mechanisms, including activation of p53 and binding to and inhibition of the ribosomal chaperone nucleophosmin/B23 (NPM) [182,183].

Recent studies suggest that accelerated senescence is a state of continuous cell proliferative signaling in the presence of a cell cycle blockade [184–187]. At the center of this scheme is the mammalian target of rapamycin (mTOR), which acts as a hub for many of the signaling pathways involved in cell growth, proliferation and homeostasis [188]. mTOR inhibition by rapamycin attenuates the increase in senescence-associated β-galactosidase activity in several models of cellular senescence [184–187]. Indeed, rapapmycin is a well-established inducer of autophagy, which may directly impede accelerated senescence. Furthermore, a recent study by Iglesias-Bartolome demonstrated that rapamycin blocks radiation-induced epithelial stem cell senescence via p16/ink4a and substantially reduces subsequent mucositis in a murine model of head/neck radiation injury [12]. This study also provides evidence that mTOR activation is critical for IR-induced accelerated senescence.

Exposure to IR has been shown to cause some levels of accelerated senescence in almost all types of cells, whether transformed, immortalized or normal cells [27]. It appears that accelerated senescence is the default response of some cell types to IR where accelerated senescence occurs at lower doses than those required for the induction of apoptosis or necrosis in same cells [27]. A variety of cancer cell types contain mutations to avoid cellular senescence. Mutations in Rb, p53, and p16 have been demonstrated to lead to continued proliferation in the presence of DNA damage or oxidative stress, and ectopic expression of these proteins can induce spontaneous senescence [181,189,190]. Furthermore, the p16INK4a pathway appears to be a back-up mechanism for the p53/p21waf1 pathway as p53-deficient fibroblasts exposed to IR to induce senescence accumulate p16INK4a, but not p21 [190].

### 3.5. Radiation Effects in Bystander Cells

For many years, a dogma in radiation biology posited that manifestation of biological effects of IR is solely due to direct actions on irradiated cells. This classical target theory asserts that cellular damage is due to the deposition of energy for the ionization of biological macromolecules and/or the interactions of generated reactive oxygen species with biological molecules [191]. However, an accumulating body of evidence has established non-targeted (bystander) effects as a *bona fide* response to IR exposure [191–193]. The radiation bystander effect is broadly defined as the induction of biological effects in cells that do not directly absorb radiation including cells that are in close proximity to irradiated cells or, in the case of partial body irradiation, cells in distant organs [193,194]. The significance of the radiation bystander effect is increasingly appreciated as a long-term side effect of radiation exposure. Research indicates that the bystander effect of radiation can be positive or negative [194]. Similar to findings regarding the direct effects of radiation, the bystander effects of radiation are dependent upon the LET, dose rate, total dose and specific radiosensitivity of the cells [194]. The negative effects of radiation on bystander cells include accelerated senescence, necrosis, and apoptosis, each contributing to decreased clonogenicity [195,196]. In contrast, radiation can induce increased proliferation of tumor cells in some settings [194].

Early evidence for the bystander phenomenon originated from experiments utilizing cell culture medium transfer from irradiated cells to non-irradiated cells or the co-culture of irradiated cells with non-irradiated cells [196,197]. Cell culture studies have demonstrated that bystander cells can accumulate DNA damage following exposure to signals from irradiated cells, as evidenced by the development of micronucleus formation, DSB, and sister chromatid exchanges [195,196,198–201]. DNA effects were demonstrated in non-transformed, non-immortalized bystander cells as well as in some transformed cell types [195,196,201]. Research has revealed potential roles for direct cell-cell communication through gap junctions between irradiated cells and bystander cells, as well as the release of soluble factors from irradiated cells to induce bystander effects. A range of extracellular molecules released from irradiated cells has been implicated in bystander effects, including ROS and a variety of secreted factors such as transforming growth factor-β1 (TGF-β1), tumor necrosis factor-α, and/or cyclooxygenase-2 [193].

Paradoxically, although radiation often has lethal effects on many types of bystander cells, some cells exhibit increased growth as an indirect effect of radiation [194]. Increased proliferation has been observed in normal liver epithelial cells and non-transformed fibroblasts, as well as in several transformed cells [53,202]. Proliferation in bystander cells has been linked to the release of soluble factors into the medium [53,194]. Nitric oxide and TGF-β1 have been identified as potential agents mediating bystander proliferation in some non-transformed cells [203,204]. Another recent study of the effects of radiation-induced tumor cell apoptosis on overall tumor proliferation identified the release of prostaglandin E2 as a proliferative factor [53]. The phenomenon linking tumor cell apoptosis induction to out-of-field tumor cell proliferation has been termed a “Phoenix Rising” pathway [53]. DNA alterations combined with the proliferative effects in bystander cells may together be responsible for the nonlinear threshold model for radiation-induced carcinogenesis [194].

Studies of animal models have attempted to identify bystander effects and mechanisms *in vivo* [194]. Partial lung irradiation in rats demonstrated that regions of the lung outside of the radiation field exhibited DNA damage, albeit at lower levels than areas of the lung exposed to direct irradiation [10,205]. However, the DNA damage in out-of-field areas of the lung was attributed to the generation of ROS by activated inflammatory cells, and the damage could be suppressed by superoxide dismutase or ROS scavengers [10,197]. In mice exposed to unilateral X-irradiation, DNA damage was demonstrated in skin tissue more than 1 cm outside of the radiation field [206]. However, in radiation skin injury, excessive delayed tissue damage can be attributed to the activation of resident mast cells and other inflammatory cells [207]. Thus, the interpretation of *in vivo* out-of-field radiation effects may be complicated by radiation-induced inflammation that can induce tissue damage independently from bystander mechanisms.

## 4. Conclusions

Radiation-induced toxicity in mammalian cells involves various modes of cell death including apoptosis, necrosis, and autophagy, as well as accelerated senescence. Complex signaling events are induced in these modes of cell death and the cellular response is likely dependent upon the radiation exposure as well as the myriad aspects of the cellular context. The precise mechanisms for the biological selection of a specific mode of cell death following IR exposure have not been unequivocally established. Additionally, the mechanism(s) by which radiation induces death in cells directly affected by radiation are likely different from the mechanism(s) by which bystander cells undergo cell death. Knowledge of the mechanisms for radiation-induced damage in the context of cell death modes may provide insights into the development of more effective therapeutic countermeasures and interventions for the mitigation of radiation associated pathologies.
